# Antioxidant, cytotoxic, and genotoxic potentials of the gum of *Ferula gummosa* Boiss on PC-3 cells

**DOI:** 10.22038/AJP.2022.21605

**Published:** 2023

**Authors:** Fatemeh Eizadifard, Majid Tafrihi, Maryam Mohadjerani

**Affiliations:** *Department of Molecular and Cell Biology, Faculty of Basic Sciences, University of Mazandaran, Babolsar, Iran*

**Keywords:** Ferula gummosa, PC-3 cells, Cytotoxicity, Antioxidant potential, Caspase-3/7 activity

## Abstract

**Objective:**

*Ferula gummosa *Boiss is a well-known Iranian endemic plant that has been used in Iranian traditional medicine against various diseases. This study aimed to evaluate the antioxidant and cytotoxic capacity of *F. gummosa* gum on prostate cancer PC-3 cells.

**Materials and Methods:**

In this study, we evaluated the total phenolic and flavonoid contents, and antioxidant potentials of the gum. The MTT experiment was conducted to assess the cytotoxic potential of the gum on PC-3 cells. The clonogenic, micronucleus formation, and acridine orange/ethidium bromide staining methods were used to evaluate the survival and proliferation of PC-3 cells. DNA degradation and caspase 3/7 activity evaluations were used to assess apoptosis. The inhibitory effect on the migration of PC-3 cells was examined by *in vitro* wound-healing experiment.

**Results:**

Total phenolic and flavonoid contents, and antioxidant potential of the gum were 9.22 mg of gallic acid equivalent (GAE)/g, 3.6 mg of quercetin equivalents (QE) /g of the extract, and 13 μg/ml, respectively (compared to gallic acid and quercetin, respectively) (p<0.05). The IC_50_ value was 9.14 µg/ml for 48 hours (compared to non-treated cells) (p<0.01). The pattern of DNA degradation, and caspase 3/7 activity levels (compared to non-treated cells) (p<0.05) proposed decreased cell viability that may be due to apoptosis induction. Microscopic observations revealed nuclear condensation, a significant increase in the formation of micronuclei, and inhibition of forming colonies (compared to non-treated cells) (p<0.01) in PC-3 cells treated with 8 and 10 μg/ml of the gum. Wound-healing assessment showed the migration suppression potentials of the gum (compared to non-treated cells) (p<0.05).

**Conclusion:**

These results indicate that *F. gummosa* has considerable antioxidant and cytotoxic properties that can make it a good nominee for subsequent investigations.

## Introduction

Cancer is uncontrolled cell growth and division. As the World Health Organization (WHO) estimated in 2015, in many countries (91 of the 172 studied countries), cancer is a leading cause of death (Bray et al., 2018). According to the international collaboration in cancer research (IARC) report, in 2020, more than 19 million new cancer patients, and about 10 million cancer-related deaths have been reported (Sun et al., 2021). Because of the population aging, and alterations in the distribution of main cancer-related risk factors, cancer incidence and mortality are rapidly growing, globally (Bray et al., 2018). 

Plants and plant-derived compounds have been implemented to treat different diseases for a long time. Due to their higher activity and lower toxicity, some of them have been used as anticancer drugs, directly or after some chemical modifications (Najafi et al., 2016; Kooti and Daraei, 2017). 

*Ferula* is a genus that belongs to the Apiaceae family and consists of approximately 170 species that are distributed mainly from western Asia to northern Africa. It has been documented that some of these species are endemic to Iran (Mohammadhosseini et al., 2019). Due to having sulfur-containing compounds, the essential oils or extracts prepared from the *Ferula* plants, bear a pungent odor (Mandegary et al., 2004; Mohammadhosseini et al., 2019). The extract and gum prepared from the *Ferula* genus show antioxidant, antibacterial, antimicrobial, anti-inflammatory, and anticancer activities (Mandegary et al., 2004; Gharaei et al., 2013). For example, it has been documented that essential oils extracted from *Ferula *species have remarkable antioxidant, cytotoxic, and anticancer activities (Gharaei et al., 2013; Znati et al., 2014). Also, there are several reports on the utilization of the *Ferula* genus for the treatment of different disorders such as stomachache, cholera and diarrhea, digestive diseases, diabetes, memory disorders, etc. (Bagheri and Dashti, 2015). 

*Ferula gummosa* Boiss which is called “*Barije*” in Persian is native to the western and northern parts of Iran. In addition to its applications in cosmetics and military industries (Mahboubi, 2016), *F. gummosa* has several pharmacological and therapeutic properties including antibacterial, antispasmodic, anti-inflammatory, antiepileptic, carminative, expectorant, and laxative functions. In Iranian traditional medicine, *F. gummosa* gum is utilized as an important remedy for digestive gastrointestinal infections (Fayaz et al., 2011; Mahboubi, 2016).

Prostate cancer is one of the human frequent cancers and the third cause of cancer death worldwide (Rawla, 2019). The PC-3 cell is an androgen-independent cancer cell that is used as a model for investigating classical prostate cancer (Kamalidehghan et al., 2018). A few limited studies have reported the antioxidant and cytotoxic potentials of *Ferula* extract (Gudarzi et al., 2015; Ghorbani et al., 2016; Hosseini et al., 2017). However, there is no record of the cytotoxic and genotoxic potentials of *F. gummosa* gum on PC-3 cancer cells. Therefore, the current study aimed to investigate the antioxidant potential, cytotoxicity, and genotoxic effect of *F. gummosa* gum against PC-3 cells. 

## Materials and Methods

**Preparation of** ***F. gummosa*** **gum**

The gum was collected from *F. gummosa* roots (48590-TARI), dissolved in dimethyl sulfoxide (DMSO) (50 µg/ml), and kept at -20°C till use.

**Total phenolic contents determination**

The total phenolic content (TPC) of the gum of *F. gummosa* was calculated by using the Folin-Ciocalteu procedure following the Singleton and Rossi protocol (Singleton and Rossi, 1965). Briefly, different concentrations (50 and 150 µg/ml) of the gum were added to 1.6 ml of deionized-distilled water, and then 100 µl of Folin-Ciocalteu phenol reagent was added and preserved for 3 min at 37°C. Then, 300 µl of saturated Na_2_CO_3_ (7%) was added, and the mixture was transferred to a 10-ml tube with the addition of deionized-distilled water. The concoction was held in a dark room for 120 min, and then the absorbance was quantified at 765 nm at 760 nm by an ELISA reader (BioTek, the USA). Gallic acid was used as a reference standard and the TPC is expressed as mg of gallic acid equivalents per gram of the gum on a dry basis.

**Total flavonoid contents determination**

The aluminum chloride colorimetric method was applied to analyze the flavonoid content of the gum of *F. gummosa* (Mohadjerani, 2012). The absorbance was then measured at 415 nm by using an ELISA reader (BioTek, USA). Quercetin was used as a reference Standard. 

**Evaluating the DPPH radical scavenging activity**

One milliliter of a 0.1 mM solution of 2, 2-diphenyl-1-picrylhydrazyl (DPPH) radical in methanol was added to 50 and 150 µg/ml of the gum. After vigorous vortexing and 30 min incubation in the dark room, the absorbance was measured at 517 nm by a spectrophotometer. Ascorbic acid was employed as the positive control. 

**Evaluating the Ferric reducing antioxidant power (FRAP)**

Three milliliter of the fresh FRAP reagent was added to 50 and 150 µg/ml of the gum. After vigorous vortexing and 10 min incubation at 37°C, the absorbance was measured at 593 nm using an ELISA reader (BioTek, USA). The quercetin and the aqueous solution of FeSO_4_ were used as positive control and standard, respectively. All assays were performed in triplicates.

**Cell lines and cell culture**

PC-3 and HEK-293 cell lines were acquired from the National cell Bank of Iran (NCBI, Pasteur Institute, Tehran, Iran). The cells were maintained in RPMI-1640 containing 10% fetal bovine serum (FBS) (Sigma), 1% streptomycin, and 1% penicillin (Sigma).

**MTT experiment**

In this experiment, 5×10^3^ cells were seeded in 96-well dishes (in 100 µl of growth medium), followed by treatment with 2, 5, 10, 20, 30, 40, 50, 60, and 70 µg/ml of *F. gummosa* gum for 48 hr in triplicates. The culture medium was replaced with 100 µl Phosphate-buffered saline (PBS) containing 5 µg/µl of MTT for each well. After 3 hr, the MTT solution was removed and 100 µl of DMSO was added to each well. After the complete solubilization of the dye, light absorbance was evaluated at 590 nm by an ELISA reader (BioTek, USA). 

**DNA laddering**

DNA laddering experiment was performed on 7×10^3^ PC-3 cells that were cultured in a 6-well plate and allowed to grow to 70% confluency. Cells were then treated with 4, 6, 8, 9, 10, 11, and 12 μg/ml of the gum for 48 hr. Genomic DNA was extracted by using the salting-out method (Naeimi et al., 2022). 

**Acridine orange/Ethidium bromide (AO/EtBr) staining**

PC-3 cells grown on gelatin-coated coverslips were treated with 8 and 10 µg/ml of gum in a 6-well plate for 48 hr. The cells were then washed with PBS and fixed in a 50:50 v/v of cold acetone-methanol solution for 20 min at -20°C. Cells on the coverslips were covered with a 100 µg/ml AO and 100 µg/ml EtBr (Sigma) solution for 30 min. The coverslips were washed twice in PBS, air-dried, and then, viewed under a UV microscope (Nikon). 

**Clonogenic assay**

The clonogenic assay is an *in vitro* cell survival investigation method based on growing a single cell into a colony comprising at least 50 cells (Franken et al., 2000). In this assay, 50 cells of PC-3 were seeded in a 24-well plate in triplicate. After 3 hr when the cells attached to the substratum, they were treated with 4, 6, 7, 8, 9, 10, and 12 µg/ml of the gum for 7 days. Fixation was then conducted by using methanol-acetic acid (7:1) for 15 min followed by staining with crystal violet (0.5%) and after 2 hr, rinsing with tap water was performed. Air-dried slides were visualized by a stereomicroscope (Olympus, Japan). 

**Micronucleus evaluation**

*In vitro* micronucleus assay was conducted as described by Fenech (2000) with some minor modifications (Fenech, 2000). In this experiment, 1.2×10^4^ cells were cultured in 24-well plates in triplicates and allowed to grow to 70% confluency. Cell treatment was then performed with 4, 6, 7, 8, 9, 10, and 12 µg/ml of the gum. After 15 hr, the cells were treated with 4 µg/ml of cytochalasin B for 28 hr to arrest cytokinesis. After harvesting, the cells were placed in a cold 0.57% KCl solution followed by fixation with cold methanol: glacial acetic acid (6:1) mixture, and air-drying for 48 hr. The staining process was performed by using 15% Giemsa in a PBS buffer (pH 7.4) for 15 min and allowed to air-dry for 24 hr. The slides were then visualized and scored by light microscopy at 400× magnification. 

***In vitro*** **wound-healing experiment**

In this experiment, 7×10^3^ PC-3 cells were cultured in 6-well plates in triplicates, to grow to confluence. All steps were performed as described in our previously published study (Naeimi et al., 2022). 

**Detection of caspases 3/7 activity**

The detection of caspase-3/7 activity was performed according to the manufacturer’s instructions (Kiazist, Iran), as described in our previously published study (Naeimi et al., 2022).

**Statistical analysis**

The data are shown as the means±standard deviations from the three replicates. Results were analyzed by using the SPSS software (IBM. Statistic25). p-values <0.05 were considered significant. IC_50_ was calculated by Excel (Office 2016) and the GraphPad Prism software version 8 for Windows (GraphPad Software, La Jolla, California, USA, http://www.graphpad.com/). Measuring the wound surfaces was performed by using the ImageJ software (NIH, Bethesda, MD, USA).

## Results

**Total phenolic and flavonoid content and antioxidant activity**

The total phenolic content of the dried extract of the gum of *F. gummosa* was measured as 0.09 µg of gallic acid equivalent (GAE) per µg of the extract. The average total flavonoid content was 0.2 µg of quercetin equivalents (QE) per µg of dried extract of the gum of *F. gummosa*. The radical cleansing activity of the gum of *F. gummosa* and ascorbic acid was evaluated by the DPPH method. IC_50_ value of *F. gummosa* and ascorbic acid was respectively 13 and 14 mg/ml.

The antioxidant potential of the gum was examined by the FRAP assay. The antioxidant activity was stated as the of antioxidant concentrations with ferric reducing ability equivalent to that of 1 µM of FeSO_4_. Our results demonstrated that the ferric reducing power of the extract and quercetin were 16 and 1.2 µM of FeSO_4, _respectively. 

**Effect of** ***F. gummosa gum*** **on the viability of PC-3 cells**

**Figure 1:**
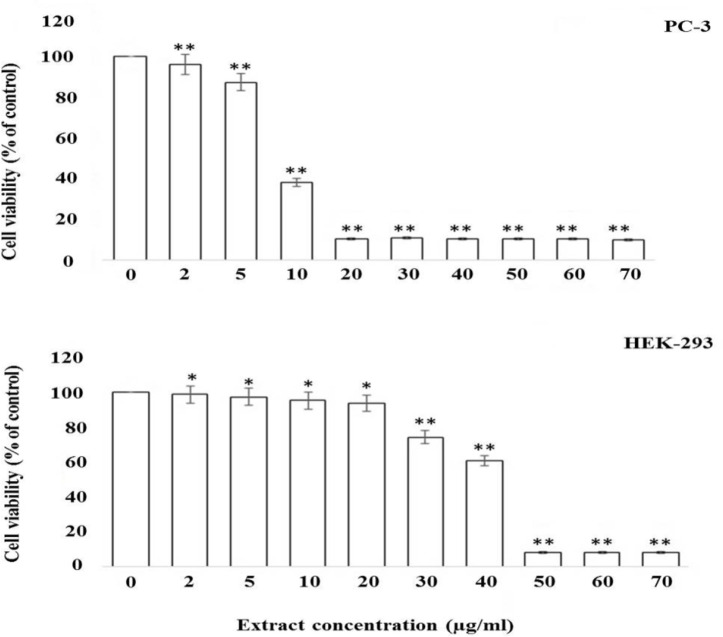
The gum of *F. gummosa* reduces the viability of PC-3 and HEK-293T cell lines. PC-3 and HEK-293T cells were seeded in 96-well plates and allowed to grow to 70% confluency. The cells were then treated with different concentrations of the gum of *F. gummosa* for 48 hr and then the viability of cells was measured by the MTT assay. The data shown are mean±SD of three separate experiments in which each treatment was repeated in 10 wells (*p<0.05 and **p<0.01 vs. control).

Figure 1 shows that the treatment of cells with 2 and 5 µg/ml of the gum of *F.* *gummosa* resulted in a minor reduction in cell viability, but treatment with 10 µg/ml or higher concentrations of the gum led to a remarkable reduction in the cell viability. According to Figure 1A, the concentration of 10 µg/ml of the gum resulted in a 60% reduction in cell viability, but there was no significant difference between 20 µg/ml and higher concentrations. The IC_50_ value of the gum for the PC-3 cell was estimated 9.14 µg/ml. Unfortunately, because the normal human prostate cell line was not available, we used the HEK-293T cells as the substitution. We found that the gum has less effect on the HEK-293T cells viability as a normal cell line (IC_50_= 38.18 µg/ml) (Figure 1). 

**Figure 2:**
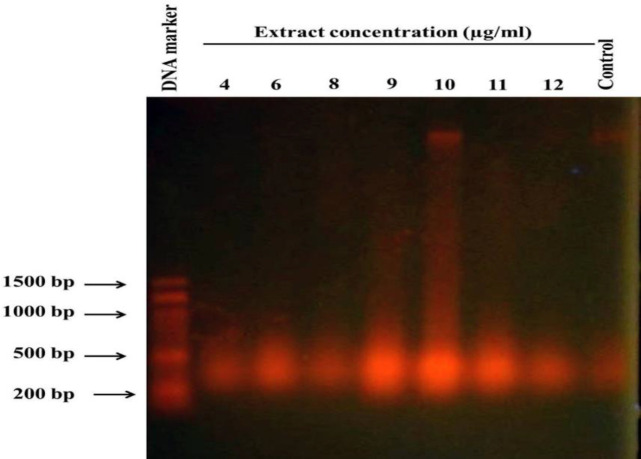
The gum of *F. gummosa* induces DNA degradation in PC-3 treated cells. PC-3 cells were treated with 4, 6, 8, 9, 10, 11, and 12 µg/ml of the gum. The DNA extracted from each group was electrophoresed on 1% agarose gel followed by ethidium bromide staining. The control lane represents non-treated cells.

We then performed DNA fragmentation experiments to study whether the gum induces apoptosis or not. PC-3 cells were treated with 4, 6, 8, 9, 10, 11, and 12 µg/ml of the gum for 48 hr, and then genomic DNA extraction was performed. For electrophoresis, similar amounts of DNA from any sample were loaded on 1% agarose gel followed by ethidium bromide staining. As Figure 2 shows, DNA degradation was visible as a smear and a classic DNA ladder was never detected. However, smear intensity increased with concentration up to 10 µg/ml of the gum. It is inferred that the disappearance of the smear pattern in cells treated with higher concentrations including 11 and 12 µg/ml (Figure 2), is due to a significant decrease in the number of viable cells.

***F. gummosa gum*** **induces nuclear condensation**

**Figure 3:**
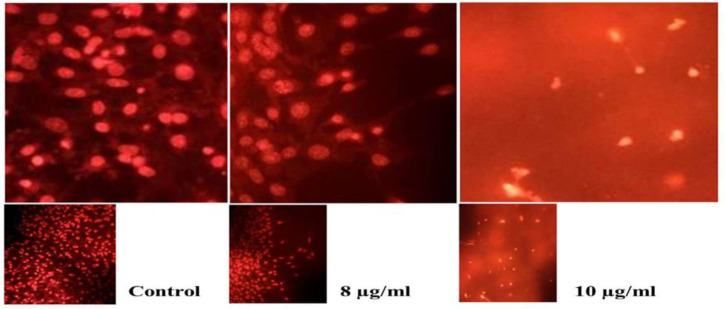
The gum of *F. gummosa* induces nuclear condensation in PC-3 treated cells. PC-3 cells were seeded in 6-well plates and treated with different concentrations of the gum for 48 hr. PC-3 cells treated with 8 and 10 µg/ml of the gum showed nuclear condensation and fragmentation. No such changes were observed in non-treated (control) cells.

For the next step, PC-3 cells were processed with various gum concentrations*,* and then cells were stained with Acridine orange/Ethidium bromide (AO/EB) followed by visualization with a fluorescence microscope. As Figure 3 shows, no serious change was identified in the nucleus of non-treated cells as a control group, but cells treated with concentrations near the IC_50_ including 8 and 10 µg/ml of the gum showed nuclear condensation (Figure 3). 

**The** ***gum of F. gummosa*** **inhibits the clonogenic capacity of PC-3 cells**

Clonogenic analysis is commonly used to investigate the survival of treated cancer cells. Figure 5 represents the clonogenic survival of PC-3 cells processed with varying concentrations of *F. gummosa *gum. As results show, the colonies decreased in a concentration-associated mode. After counting clones, PE and the surviving fraction (SF) were computed by using the following equations:

PE= $\frac{\mathrm{\# of colonies formed}}{\mathrm{\# of cells seeded}}\times 100\%$

SF= $\frac{\mathrm{\# of colonies formed after}}{\mathrm{\# of cells seeded}\times \mathrm{PE}}\times 100\%$

**Figure 4:**
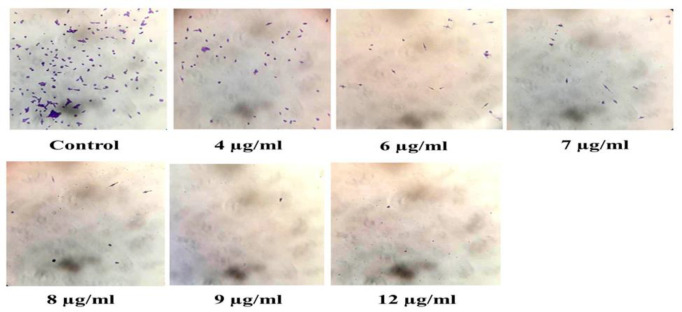
The gum of *F. gummosa* inhibits the colony formation of treated PC-3 cells in a concentration-dependent manner. PC-3 cells were seeded in a 24-well plate and after they were attached to the dishes, they were treated with different concentrations of the gum for 7 days. After fixation and staining, the colonies were checked. The data shown are mean±SD of three separate experiments in which each treatment was repeated in 10 wells (*p<0.05 and **p<0.01 versus control).

For PC-3 cells, PE was about 60%, and the SF was calculated as 1.7, 1.2, 0.95, and 0.58 for concentrations of 8, 9, 10, and 12 µg/ml of the gum, respectively (Figure 4).

***F. gummosa gum*** **induces micronucleus establishment in PC-3 cells**

**Figure 5:**
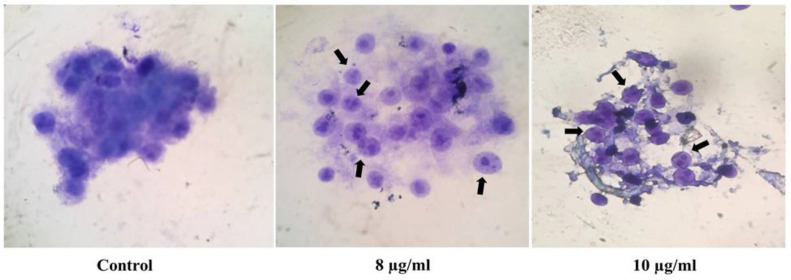
The gum of *F. gummosa* induces micronuclei formation in PC-3 cells. PC-3 cells were cultured in a 24-well plate and allowed to grow to 70% confluency. The cells were then treated with sublethal and/or lethal concentrations of the gum, showed a higher frequency of micronuclei or binucleated cells.

It was concerning to know that *F. gummosa* gum has any genotoxic consequence on the cytokinesis of PC-3 cells. Microscopic examinations revealed that PC-3 cells treated with 2, 4, and 6 µg/ml of the gum were not significantly different from the control group, but compared to the control (untreated) cells, the prevalence of binucleated cells or micronuclei in cells processed with 8 and 10 µg/ml of the gum was considerably increased (Figure 5). 

***F. gummosa gum*** **prevents PC-3 cells invasion**

**Figure 6:**
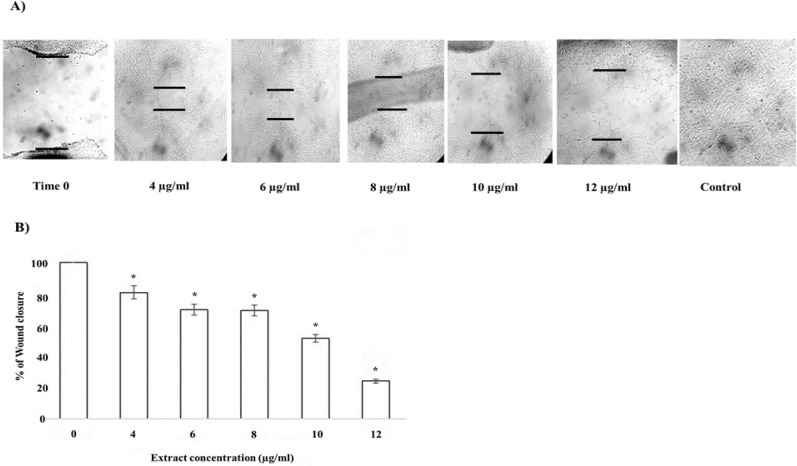
The gum of *F. gummosa* inhibits the invasion of PC-3 cells. A) Wound healing graphs of PC-3 cells treated with different concentrations of the gum. The black short lines represent the border of the wounds. B) The wound closure values of the sheets treated with different concentrations of the gum. The data shown are mean±SD of three separate experiments in which each treatment was repeated in 10 wells (*p<0.05 *vs.* control).

After investigating the cytotoxic and genotoxic activities of *F. gummosa *gum*,* we decided to assess the inhibitory effect of the gum on the invasion of PC-3 cells by using the wound-healing procedure. As shown in Figure 6A, compared to the untreated (control) cells (100%), the wound repair rate decreased in a dose-dependent fashion. As we anticipated, the wound repair rate for cells processed with high concentrations of the gum was much lower than that of non-treated cells, in which the area covered was 100% close to the original gap size after 48 hr (48.63 and 75.90% for cells treated with 10 and 12 µg/ml of the gum, respectively) (Figure 6B).

**Caspases 3/7 Activity**

**Figure 7:**
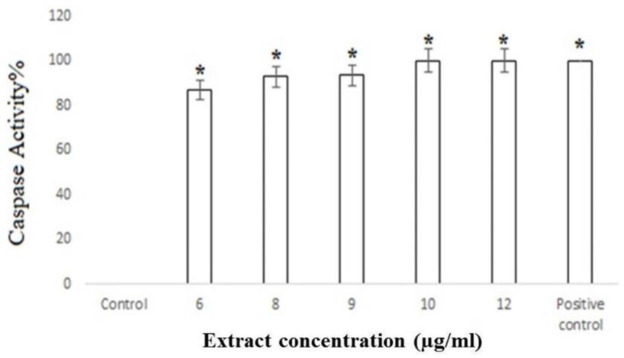
Caspases 3/7 activity in PC-3 cells treated with different concentrations of the gum of *F. gummosa*. PC-3 cells were seeded in 6-well plates and treated with different concentrations of the extract for 48 hr. According to the kit instructions, the levels of caspases 3 and 7 activities were evaluated. The positive control contained the substrate of caspases 3 and 7. The shown data are mean±SD of three independent experiments (*p<0.05* vs. *control).

The activation of caspases 3/7 was evaluated in cells treated with various concentrations of the gum of *F. gummosa*. Figure 7 shows that compared to that of nontreated cells (0%) caspase-3/7 activity in treated cells significantly increased several folds in a concentration-dependent fashion at 6 to 10 μg/ml of the gum (Figure 7).

## Discussion

Chemotherapy, radiation therapy, and surgery are still usual cancer treatment approaches that could be effective, especially for primary solid tumors, but each of them has side effects (Rockwell et al., 2009). Due to their invasive and non-selective manner, these approaches kill normal and cancer cells (Baldo and Pham, 2013). 

Plants represent an important source with amazing chemical diversity for the development of new medicines (Rockwell et al., 2009). Some of them contain chemical compositions with noticeable cancer-fighting potential and have been found, purified, and used for the cancer prevention and treatment (Mans et al., 2000; Hossein and Ghorbani, 2015). 

In this study, we investigated the antioxidant and anti-cancer potentials of the gum of* F. gummosa *gum, which grows locally in Iran. 

Phenolic and flavonoid compounds are well-known plant metabolites that play important roles in maintaining human health and healing and preventing many illnesses including cancer (Tungmunnithum et al., 2018). The hydroxyl groups in these compounds are responsible for free radical cleansing and antioxidant functions (Aryal et al., 2019). In this study, we found that the gum of *F. gummosa* contains remarkable whole phenolic and flavonoid constituents that are liable for its significant antioxidant and free radical cleansing activities. Plants of the *Ferula* genus that are distributed in some countries in Asia and Africa show remarkable antioxidant properties (Sahebkar and Iranshahi, 2010). It has been publicized that the methanolic extract of *F. szovitsiana *and *F. microcolea* shows striking free radical cleansing and antioxidant functions (Dehghan et al., 2007; Amiri, 2014). Ebrahimzadeh announced that the IC_50_ value for the DPPH radical-cleansing function of the hydroalcoholic extract of the *F. gummosa* was 579.6 µg/ml, while our study revealed the gum shows greater antioxidant properties (IC_50_ 130 µg/ml) (Ebrahimzadeh et al., 2011). According to our results, the gum of *F. gummosa* shows significant antioxidant properties and could be a good nominee for subsequent analyses. 

According to the MTT results, the sensitivity of cancer cells to the *F.* *gummosa* gum was significantly higher (IC_50_ 9.14 µg/ml) than that of HEK-293 normal cells (IC_50_ 38.18 µg/ml) (Figure 1). By comparing the cytotoxic properties of *F. gummosa* with some known and clinically-used anticancer drugs against PC-3 cells including bortezomib (200 µg/ml), etoposide (1730 µg/ml), and irinotecan (12000 µg/ml), we found that *F. gummosa* was much more potent and cytotoxic (Aras and Yerlikaya, 2016).

The presence of a highly toxic compound to PC-3 in *F. gummosa* gum or two or more compounds that work synergistically against PC-3 cells are the two main possibilities for its low IC_50_ value. Studies have shown that *Ferula *species are rich in monoterpene hydrocarbons that suppress inflammation and carcinogenesis at initiation and/or progression stages (Utegenova et al., 2018). Chemical analyses have shown that monoterpenes including α-pinene and β-pinene are the most abundant constituents of the essential oil prepared from distinct parts of *F. gummosa* (Sahebkar and Iranshahi, 2010; Najafabadi and Naghavi, 2018). Mounting evidence confirms the synergism mode for some phytochemicals that have suppression potentials against cancer cell growth and division (Liu, 2004; Wright et al., 2007). It can be considered an experimental or clinical approach to suppressing cancer cell growth because the synergistic effect minimizes the aftereffects of a single molecule. 

The DNA laddering outcomes revealed that the gum reduces cell livability due to the induction of apoptosis. DNA fragmentation and apoptosis induction emerged at a concentration of 9 µg/ml of the gum and persisted in a concentration-associated mode (Figure 2). Therefore, we did not observe a noticeable decrease in cell viability at two lower concentrations of the gum (4 and 6 µg/ml), so no obvious DNA fragmentation was observed. AO/EtBr dual staining results showed that cells treated with concentrations near IC_50 _(8 and 10 µg/ml) led to nuclear condensation (Figure 3). 

The clonogenic assay as a slow-acting test revealed that the gum of *F. gummosa* has potential anticancer activity and limits the multiplication of PC-3 cells in a concentration-associated mode (Figure 4). 

Micronucleus induction is considered a genotoxic factor and a factor for chromosome damage (Fenech, 2000). The results of micronucleus formation experiments indicated that the gum of *F. gummosa* is genotoxic, as lower concentrations of the gum (2, 4, and 6 µg/ml) had no genotoxic consequence on PC-3 cells, but concentrations near IC_50 _(8 and 10 µg/ml) induced micronucleus formation (Figure 5). Although several reports are indicating the anti-genotoxic properties of some plant-derived compounds, it has been documented that some plant extracts or compounds have micronucleus induction potentials (Cea et al., 1983; Aithal et al., 2009; Oyeyemi et al., 2015). 

As a primary investigation, we performed the *in vitro* wound-healing experiment to study the inhibitory potentials of *F. gummosa* gum on migration and invasion of PC-3 cells. Although the sublethal doses (2 and 5 µg/ml) of *F. gummosa* gum did not have a significant effect on the cell livability, higher concentrations of the gum led to a noticeable reduction in wound healing rate (Figure 6). The healing process of the wound in a cell monolayer is dependent on cell multiplication and migration (Grada et al., 2017). As we detected a minor inhibitory effect of the gum on cell viability at concentrations of 2 and 5 µg/ml, no considerable reduction in wound closure was observed in cells treated with these concentrations. We concluded that the reduction in wound closure at these concentrations might be due to a decrease in cell invasion.

Induction of apoptosis is an ideal strategy in cancer treatment studies. Caspase 3 and caspase 7 are final executioners that are both involved in cell death, regardless of the death-inducing stimulant (Walsh et al., 2008). Gharaei et al showed that petroleum ether extract of *F. gummosa* leaf and flower derives apoptosis in the AGS cell line (Gharaei et al., 2013). In this study, DNA laddering and nuclear staining demonstrated that treated cells show some signs of apoptosis. Caspase activity assay results indicated that the gum of *F. gummosa* enhanced the activity of executioner caspases 3/7 in the extrinsic pathway.

Therefore, this is the first study on the anticancer potential of the gum of *F. gummosa*, the effective compound(s) and underlying mechanisms have not been studied, yet. Analyzing the chemical composition of the gum of *F. gummosa* and recognizing the responsible molecule(s) for its anticancer function may explain the underlying mechanisms. In the next step, we are planning to isolate the components of *F. gummosa* to recognize the active and effective components against tumor cells.

## Conflicts of interest

The authors have declared that there is no conflict of interest.
